# Hemodynamic Bedside Monitoring Instrument with Pressure and Optical Sensors: Validation and Modality Comparison

**DOI:** 10.1002/advs.202307718

**Published:** 2024-04-22

**Authors:** Matti Kaisti, Tuukka Panula, Jukka‐Pekka Sirkiä, Mikko Pänkäälä, Tero Koivisto, Teemu Niiranen, Ilkka Kantola

**Affiliations:** ^1^ Department of Computing University of Turku, Faculty of Technology Vesilinnantie 5 Turku 20500 Finland; ^2^ Department of Internal Medicine University of Turku Kiinamyllynkatu 4‐8 Turku 20521 Finland; ^3^ Division of Medicine Turku University Hospital Kiinamyllynkatu 4‐8 Turku 20521 Finland

**Keywords:** blood pressure, hemodynamics, instrument, photoplethysmography, sensor

## Abstract

Results from two independent clinical validation studies for measuring hemodynamics at the patient's bedside using a compact finger probe are reported. Technology comprises a barometric pressure sensor, and in one implementation, additionally, an optical sensor for photoplethysmography (PPG) is developed, which can be used to measure blood pressure and analyze rhythm, including the continuous detection of atrial fibrillation. The capabilities of the technology are shown in several form factors, including a miniaturized version resembling a common pulse oximeter to which the technology could be integrated in. Several main results are presented: i) the miniature finger probe meets the accuracy requirements of non‐invasive blood pressure instrument validation standard, ii) atrial fibrillation can be detected during the blood pressure measurement and in a continuous recording, iii) a unique comparison between optical and pressure sensing mechanisms is provided, which shows that the origin of both modalities can be explained using a pressure‐volume model and that recordings are close to identical between the sensors. The benefits and limitations of both modalities in hemodynamic monitoring are further discussed.

## Graphical abstract

Illustration of a bedside hemodynamic monitoring system capable of spot‐continuous measurements of blood pressure and continuous analysis of rhythm. The system is housed in a form factor similar to that of a common pulse oximeter. The combination of optical and pressure sensors allows for comprehensive bedside monitoring with minimal device count.

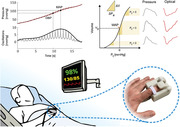

## 1 Introduction

Non‐optimal blood pressure (BP) continues to be a major risk factor for cardiovascular diseases (CVD), leading to more than 10 million deaths and 212 million years of healthy life lost each year.^[^ ^1^ ^]^ At the same time, hypertension is the most common preventable risk factor for CVD.^[^ ^2^ ^]^ Diagnosed hypertension can be effectively controlled with medication and lifestyle changes. The condition, however, is mostly asymptomatic and less than 20% of people with hypertension have it under control.^[^ ^3^, ^4^, ^5^ ^]^

Hypertension currently affects more than a billion people worldwide,^[^ ^5^ ^]^ and the burden is disproportionately distributed in low‐income regions. Despite several initiatives to limit the burden of hypertension, its global prevalence and adverse effects are increasing.^[^ ^5^, ^6^ ^]^ Proper control of BP has been shown to significantly reduce cardiovascular morbidity and all‐cause mortality associated with hypertension.^[^ ^7^ ^]^

In addition, close monitoring of BP is required in the bedside setting, such as in hospital wards, where patients can experience hypotension possibly leading to inadequate perfusion of vital organs, which can result in organ damage or failure.^[^ ^8^ ^]^ Therefore, accurate and continuous monitoring of BP is needed for the timely detection and management of hypotension and ultimately for improving patient outcomes.

In recent years, several studies have investigated the possibility of pulse propagation methods, such as pulse transit time (PTT) or pulse arrival time (PAT) for indirect but continuous BP monitoring.^[^ ^9^ ^]^ Such indirect methods still typically require frequent calibration and clinical validation studies are rare^[^ ^10^ ^]^ and standards do not recommend their use. Another line of research has focused on demonstrating the possibility of measuring BP without any contact using camera‐based detection.^[^ ^11^, ^12^ ^]^ The technical challenges, however, for non‐contact BP estimation are even greater than those in pulse propagation methods. Other efforts include miniaturization of the BP instruments^[^ ^13^ ^]^ using the well‐known oscillometric technique. An oscillometric response can be recorded with a pressure cuff, with an optical photoplethysmography (PPG) sensor^[^ ^14^ ^]^ or via a finger pressing method.^[^ ^13^, ^15^, ^16^, ^17^ ^]^

**Figure 1:**
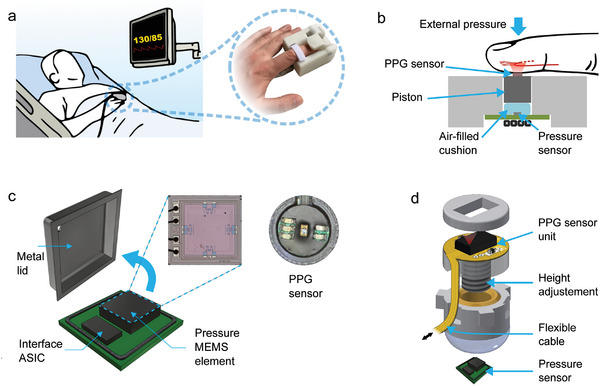
Overview of the instrument. a) An example of the bedside monitoring use case and a picture of the instrument. b) Cross‐sectional view of the sensor setup. Slowly increasing and decreasing external pressure is exerted on top of the fingertip. Sensory responses from both the pressure and optical sensors are read from the bottom of the fingertip. c) Barometric pressure sensors element with the metallic cover removed. d) The piston comprises PPG electronics (on top) and an air cushion (bottom), which rest on the modified barometric sensor. The parts are connected by a threaded shaft, enabling height adjustment.

In this study, we present results from compact instrument for measuring hemodynamics at the bedside designed to have similar form factor as in commonly used pulse oximeters (**Figure** **1**a). The developed technology is presented and analyzed in three different embodiments, all sharing the same fundamental technology. Two embodiments are the first and second prototypes of the same device, where the second one is a miniaturized version. The third device version shares the same fundamental technology, but additionally includes a PPG sensing unit not used in the first and second embodiment. The sensing structure is illustrated in Figure 1b–d. The developed instrument consists of a pressure sensor, optionally a PPG unit, and a motor that applies external compression force to the fingertip, as shown in Figure 1b.^[^ ^13^, ^17^ ^]^ To measure BP, the user places their fingertip on top of the sensor, and the compression force is then gradually increased. The recorded pressure and PPG signal is processed to extract the oscillometric envelope, from which the BP values can be estimated. We also describe the operating principle of this technology, validate the blood pressure measurement accuracy in two clinical studies. In addition, we demonstrate the capability to measure atrial fibrillation and compare pressure and optical modalities in hemodynamic monitoring.

## 2 Results

### 2.1 Operating Principle

**Figure 2:**
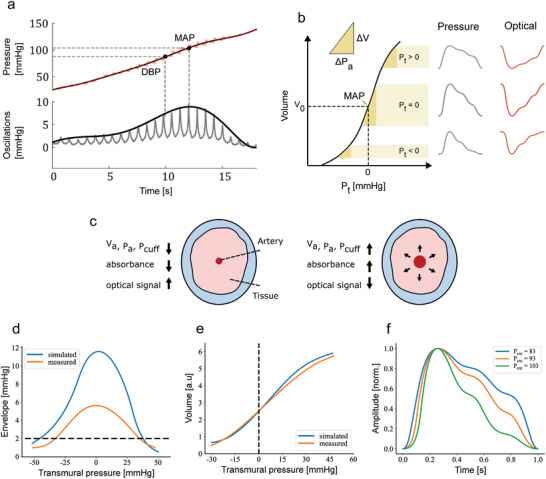
Overview of the measurement technique. a) Example of measured oscillometric response obtained after fitting and envelope on the processed pressure ramp signal. The maximum point on the bell‐shaped curve indicates the point of the MAP in the pressure ramp from which DBP and SBP are calculated. b) Illustration of pressure‐volume relationship explaining the oscillometric response. The steepness is highest around MAP i.e., the volume changes are largest per unit change in transmural pressure (*P*_*t*_). c) Simplified cross‐sectional view of measurement location and the direction of change of key parameters during an arterial pulse. d) Comparison between measured and simulated and oscillogram envelopes, e) and pressure volume curves and f) further illustration of waveform shape with different external pressures, here *P*_*ext*_=93 mmHg corresponds to zero transmural pressure.

In a typical oscillometric measurement, a cuff is wrapped around the upper arm and the pressure in the cuff is gradually increased over the systolic blood pressure (SBP) until the brachial artery is occluded.^[^ ^10^ ^]^ BP can be determined by measuring cuff pressure during inflation or deflation. There is a small change in the volume of the arterial segment Δ*V*_*a*_ for each pulse causing small changes in the cuff pressure Δ*P* during each cardiac cycle. The pressure signal is a composition of a slowly decreasing/increasing pressure and Δ*P*. These oscillations are typically extracted using a high‐pass filter resulting in an oscillogram as exemplified in **Figure** **2**a. The envelope of the oscillogram is typically a bell‐shaped curve, and the maximum point closely matches the mean arterial pressure (MAP). The SBP and DBP values are computed from the envelope using a device‐specific algorithm.

The above described BP measurement principle can be explained using the pressure‐volume relationship of an arterial segment^[^ ^18^ ^]^ shown in Figure 2b. When cuff pressure (considered as external pressure *P*_*ext*_) is increased, weak oscillations are coupled to it, caused by the small pulsatile expansion of the artery within each heartbeat. The volume of the cuff remains nearly constant during the pulse, but the volume and pressure of the arterial segment (*P*_*a*_) change during the pressure pulse. With external pressure close to the SBP, the traveling pressure wave results in a modest or no change in volume, since the artery is collapsed or even occluded by the high external pressure. Similarly, as we move toward the DBP, the changes become smaller as the artery distends and becomes stiffer. Ultimately, at low external pressure, the coupling of the arterial pulsation to the sensor vanishes. The steepest part of the pressure‐volume curve is at the point where the transmural pressure, *P*_*t*_ = *P*_*a*_ − *P*_*ext*_, is zero. It is the point where the artery is flattened and most distensible and where the largest changes in arterial volume are observed. This nonlinear relationship between pressure and volume of the arteries explains the shape of the pressure oscillation envelope.

The developed instrument is based on the above explained oscillometric principle even though the pressure sensing mechanism is different. In the developed construction, a force is applied on top of the finger and the force propagates through the piston to an air‐cushion placed on top of a modified barometric pressure sensor (Figure 1b–d). The behavior of the modified pressure sensor can be explained by the mechanical construction of the sensing part of the instruments. Pressure is defined as force per unit area (*P* = *F*/*A*). The piston pressure (*P*_*piston*_) is the sum of two pressures, *P*_*aeff*_ and *P*_*ext*_. *P*_*ext*_ is the external pressure from the motion actuator applied to the finger and *P*_*aeff*_ is the effective pressure oscillation caused by the arterial pulse. The piston and cushion have nearly equal areas, and thus the pressure on the fingertip equals the pressure of the air cushion. Fixed cylinder walls prevent the cushion to expand under external pressure, resulting in an even distribution of the pressure in the cushion. Thus, the pressure exerted on the MEMS sensor equals *P*_*piston*_.^[^ ^13^ ^]^ Further details about the propagation of the external force to the barometric sensor are given in Supporting Information.

Since the optical sensor detects changes in blood volume, similar oscillometric responses can be recorded with PPG when the sensor is mounted on top of the piston. However, the optical sensor does not directly measure pressure values. To determine blood pressure, a calibration is required, for example by using a brachial cuff to relate the optical oscillometric response to pressure values. The external pressure in this setup was read using the modified pressure sensor. Therefore, the optical oscillogram responses can be explained through the same pressure‐volume relationship even if the sensing modality is different. The direction of change in key parameters during an arterial pulse are depicted in Figure 2c. In the PPG the absorbance is increased during a pulse, which leads to fewer photons traveling back to the sensor, explaining the inverted pulse waveform compared to the pressure waveform.

To further investigate and validate the behavior of the developed instruments, we used a mathematical pressure‐volume model.^[^ ^18^ ^]^ The model equations are summarized in Table S1 (Supporting Information). The measurements and model have reasonable agreement, indicating that the operation of the finger probe follows the well‐known and reliable oscillometric technique. A comparison between a typical measurement and a modeled oscillometric envelope with the same pressure values is shown in Figure 2d. The model further clarifies that the volume of the cuff or, in this case, the air cushion (*V*_*c*0_), does not affect the measured BP values, but affects the recorded oscillation amplitudes depending on the ratio of volumes in the arterial segment and the air cushion.

A typical maximum oscillation envelope amplitude of the finger probe is about 6 mmHg and about 2 mmHg for the brachial cuff when using the same envelope computation. The instrument clearly targets a smaller change in blood volume at the fingertip compared to the brachial cuff, but the significantly reduced air volume in the cushion compared to a typical cuff results in higher measured oscillation amplitudes. This results in a high signal‐to‐noise ratio and thus high‐quality recordings. In Figure 2e simulated and typical measured pressure‐volume are compared further validating the model in explaining the instrument operation. In Figure 2f the normalized pulse waveforms are compared at different external pressures. The *P*_*ext*_ = 93 mmHg equals the zero transmural pressure in this simulation. External pressure can be considered to be nearly constant during an arterial pulse, but the pulse pressure changes during a cardiac cycle around the transmural pressure. The difference in the waveform shape in positive and negative transmural pressure can be explained through the pressure‐volume graph (Figure 2b). When the external pressure is fixed in the positive *P*_*t*_ (*P*_*ext*_ = 83 mmHg) the amplitude of the waveform in the diastolic region is heightened compared to the waveform at *P*_*t*_ = 0 (*P*_*ext*_ = 93 mmHg). This is due to the concave shape of the pressure‐volume graph when moving from right to left at positive *P*_*t*_ (decreasing pressure due to decreasing *P*_*a*_) i.e., at first the change is modest and increases toward the end of pulse. At negative *P*_*t*_ the opposite is true, the change is fastest right after the systolic peak after leveling off toward the end of the pulse due to the convex shape of the curve.

### 2.2 Oscillometric Blood Pressure

**Figure 3:**
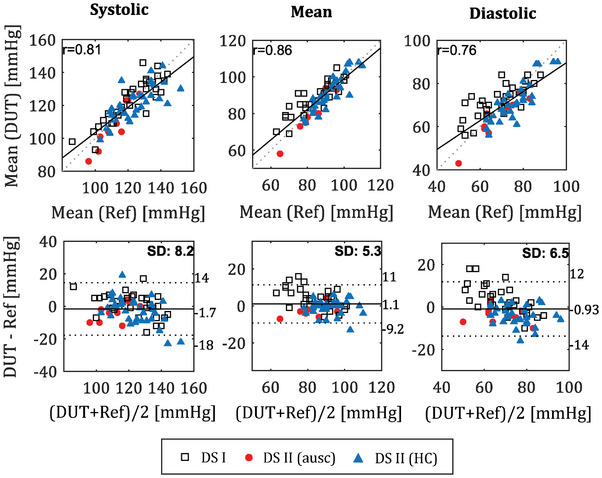
Correlation and Bland‐Altman plots for two independent clinical trials. Datapoints from dataset I (white) and dataset II (red) and dataset II HC (blue) are pooled into one set (n = 76). The correlation coefficient (r) and standard deviation (SD) are embedded in the figure.

The accuracy of BP measurements was tested in two clinical validations. In addition, a hydrostatic challenge was undertaken to broaden the measured blood pressure range, with the aim of providing a more accurate representation of higher values. This resulted in a total dataset size of 76. The measured BP ranged from 86 to 152 for SBP and from 50 to 96 for DBP. The (*mean* ± *standard deviation*) mmHg analyzed on all data for SBP, mean BP (MAP) and DBP were (−1.7 ± 8.2) mmHg, (1.1 ± 5.3), and (‐0.9 ± 6.5) mmHg, respectively. The results are summarized in **Table** **1** and **Figure** **3** with correlation and Bland‐Altman plots for DBP, MAP and SBP for the combined dataset containing dataset I and dataset II. MAP and DBP accuracies comply with the international standards for noninvasive BP monitors. The standard deviation for SBP was 8.2 mmHg which is 0.2 mmHg higher than the AAMI standard threshold of 8 mmHg.^[^ ^19^ ^]^. However, the collected data did not fully meet the requirements regarding the blood pressure range, and especially the extreme BP values are underrepresented in the dataset. Details on data collection, reference devices used, and analysis methods are given in Sections 4 and 4, respectively.

**Table 1:** Summary of BP measurement accuracy of the finger probe on two separate clinical validation trials (mean ± SD).

	SBP	DBP	MAP
Trial I	−0.9 ± 7.3	−3.3 ± 6.6	−4.3 ± 5.3
Trial II	−3.5 ± 8.4	−4.0 ± 4.4	−1.2 ± 3.9
Combined	−1.7 ± 8.2	−0.9 ± 6.5	1.1 ± 5.3

### 2.3 Atrial Fibrillation

The simultaneous time‐synchronized arterial waveform signals under controlled external pressure in dataset IIIb, including both pressure and optical sensors, were used for rhythm analysis. These continuous measurements had patients in sinus rhythm (n = 14), and in AF (n = 5).

**Figure 4:**
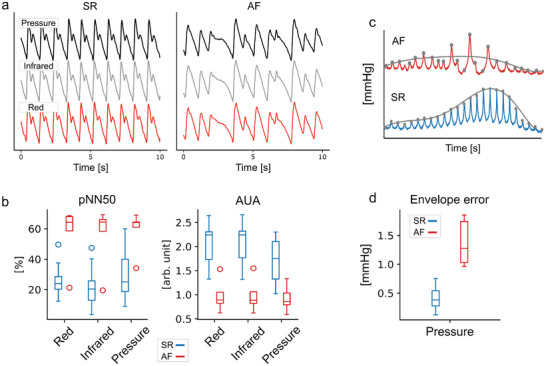
a) Typical signal traces with pressure and optical sensors from the fingertip during constant external pressure. On the left: Pulse train in sinus rhythm. On the right: Pulse train in AF. b) Feature comparison between sinus rhythm (n = 14) and atrial fibrillation, AF (n = 5). The line and whiskers in the box present the median and range of data, respectively. Irregular rhythms have lower AUAs and greater interbeat‐interval variability. c) An illustration of AF detection during oscillometric measurement. d) Comparison of an envelope error feature between AF (n = 6) and SR (n = 43) for pressure sensor.

An example of recordings after band‐pass filtering for sinus rhythm and AF for both optical and pressure sensors is shown in **Figure** **4**a. To discriminate these signals, we computed the area under autocorrelation (AUA)^[^ ^20^ ^]^ and the heart rate variability metric pNN50.^[^ ^21^ ^]^ These features demonstrate a clear difference between the two groups of patients in all channels, as shown in the box plots in Figure 4b. The ability to discriminate between the two groups is based on the detection of irregularity in the rhythm. The high quality of the recordings, mostly attributed to controlled external pressure and stable measurement conditions, allows clear discrimination between the two groups using simple signal features. In larger and less controlled trials, separability would most likely be noticeably diminished.

The detection of AF during oscillometric measurement is shown in Figure 4c. An envelope error was computed over SR (n = 43) and AF (n = 6) groups in dataset II. The developed feature can perfectly separate AF from SR, however, the amount of AF measurements is small, and as in the continuous measurements under fixed external force, the separability is expected to be lower in a larger study.

### 2.4 Comparison between Optical and Pressure Sensors

The metrics used to compare the optical and pressure channels are summarized in **Table** **2**. Each comparison is done to simultaneously measured data allowing direct evaluation between optical and pressure channels.

**Table 2:** Summary of similarity analysis between pressure and optical sensors; beat‐to‐beat detection, blood pressure accuracy and correlation analysis.

**Beat‐to‐beat detection [%]**				
	SR	SR	AF	AF
	PPV	TPR	PPV	TPR
Pressure	99.29	99.89	99.01	99.50
IR	100.00	99.95	100.00	99.50
Red	100.00	99.95	100.00	99.17

**Blood pressure (mmHg, mean ± SD)**				
Pressure	–1.2 ± 5.9			
IR	–3.1 ± 6.5			
Red	–4.8 ± 6.7			

**Correlation analysis (ρ, mean ± SD)**			
	SR	SR	AF
	waveforms	envelope	waveforms
Pressure, IR	0.88 ± 0.10	0.72 ± 0.17	0.96 ± 0.05
Pressure, red	0.87 ± 0.11	0.63 ± 0.21	0.95 ± 0.06
Red, IR	0.95 ± 0.06	0.98 ± 0.01	0.97 ± 0.04

The beat‐to‐beat detection results, positive predictive value (PPV) and true positive rate (TPR) computed over dataset IIIb, indicate high detection rates attributed to high‐quality signals. The high quality of the recordings is due to taking measurements at rest and keeping the external contact force constant. The pressure channel performance was slightly inferior compared to the optical channels.

The blood pressure estimation between the channels, measured against the common reference, reveal that pressure sensor is best at accurately estimating BP. The analysis is done using dataset IIIa. However, it has to be noted that in PPG measurement only the oscillogram can be extracted from the PPG signal and the external pressure, where the optical oscillogram reaches its maximum, is estimated from the pressure sensor. This type of a setup inherently introduces more complexity, potentially resulting in greater measurement errors. The measurement accuracy in the channels were (−1.2 ± 5.9) mmHg, (−4.8 ± 6.7) mmHg, (−3.1 ± 6.5) mmHg for pressure, red, and infrared channels, respectively, indicating that both pressure and optical sensors can be used to record oscillometric responses. These results have been comprehensively presented before.^[^ ^22^ ^]^

**Figure 5:**
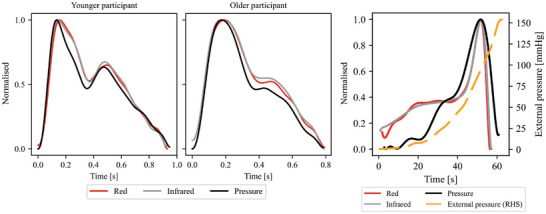
a) An example of normalized PPG and pressure pulse waveforms during one cardiac cycle for younger (male, 33 years old) and older (male, 74 years old) participants. b) Oscillometric envelopes extracted from optical and pressure signals. The maximum oscillation amplitudes occur at the same external pressure level for all of the channels.

Waveform correlation analysis between all three channels was performed for both SR and AF recordings using the dataset IIIb. Example signals are shown in **Figure** **5**a with a younger and an older participant to demonstrate that no significant differences were observed in similarity depending on age, which is a major factor affecting the waveform morphology. The correlation results are presented in Table 2. We further analyzed the similarity of the channels by calculating the correlations between the oscillogram envelopes in the dataset IIIa, also presented in Table 2. The results show, expectedly, that the red and IR channels provide nearly identical waveforms and oscillogram envelopes, whereas the pressure channel slightly deviates from the optical channels.

In conclusion, the similarity tests showed that the red and infrared channels are nearly identical. However, minor discrepancies were observed in the waveform analysis of the pressure channel compared to the optical channels. These differences can be attributed to variations in sensor bandwidth, response times, tissue‐to‐sensor coupling, and measurement errors. Furthermore, although the contact force was controlled in the dataset IIIb, slight variations in it between the sensors can still occur, leading to changes in transmural pressure, which subsequently affects the waveform. In the envelope analysis, discrepancies can also arise due to the different coupling mechanisms. The pressure sensor relies on changes in arterial volume to be detected through pressure coupling, while the optical channel registers pulses even at minimal and no contact pressures and thus can create a difference in the envelope shape in the diastolic region.

## 3 Discussion

We described the development of a miniature device capable of measuring spot BP conveniently from a fingertip in a form factor resembling that of a pulse oximeter with a capability for repeated and automated spot measurements. We validated the developed instruments with different form factors in two clinical trials, both trials providing a comparable accuracy. Furthermore, we demonstrated that pressure sensors and optical sensors can indeed be used for BP measurement. By extending our BP measurement device with a time‐synchronized optical sensor to record the oscillations, we were able to show that both sensing modalities are plausible for BP estimation and rhythm analysis, including detection of AF. The benefit of using a pressure sensing setup is that it can, similarly to a brachial cuff device, measure both external pressure (equivalent to cuff pressure in a standard digital BP monitor) and the oscillations. The optical sensor is, however, only able to measure the oscillations, and an additional sensor, although a simpler one, for the external pressure is required. The comparison between pressure and optical sensors revealed very similar pulse waveforms. Previous studies have found similar results when comparing arterial blood pressure pulse measurements using non‐invasive pressure sensors^[^ ^20^ ^]^ and photoplethysmography (PPG).^[^ ^23^ ^]^ The two optical channels, red and infrared, gave nearly identical waveforms, whereas a slightly different waveform was observed for the pressure channel. The capability for AF detection was analyzed between the sensing modalities. Both sensing modalities with controlled external pressure and in a controlled environment produced high‐quality waveforms, allowing the extraction of signal features to discriminate AF from sinus rhythm.

Blood pressure monitoring in this presented form factor allows painless measurement, in contrast to the pain associated with brachial cuff measurements for individuals with high blood pressure. The instrument also allows quick and simple measurements during night and patients do not need to be awakened for the measurement. The presented form factor would allow simultaneous measurement of oxygen saturation, thus limiting the number of monitoring devices needed. Automation would allow pseudo‐continuous BP measurement, that is, taking automated measurements periodically for BP trend tracking and analysis. For example, nocturnal hypoxemia, which is seen as a decrease in peripheral oxygen saturation during sleep, a marker of chronic obstructive pulmonary disease^[^ ^24^ ^]^ and obstructive sleep apnea could be monitored. Nocturnal BP has also been considered a sensitive predictor of cardiovascular morbidity and mortality, where both mean BP and nocturnal dipping are risk factors for CVDs.^[^ ^25^ ^]^

The technology has benefits over existing instruments, as discussed above, but also some limitations. Cold fingers, for example, can lead to reduced signal quality and positioning the fingertip can sometimes be challenging. In particular, one participant had severe nail clubbing that prevented taking successful measurements. It is likely that in other similar extreme cases, that affect the correct positioning of the finger, the measurements cannot be taken reliably. Such cases, however, are rare and with correct positioning, the measurements were repeatable in this study. Additionally, the study did not fully meet the blood pressure monitor validation standard requirements on the blood pressure value distribution and rhythm analysis was based on a limited number of participants.

In conclusion, we have clinically validated our technology in two separate trials for BP monitoring and also demonstrated its potential for rhythm analysis and demonstrated similarities and differences between optical and pressure sensors for hemodynamic monitoring.

## 4 Experimental Section

### Instruments

**Figure 6:**
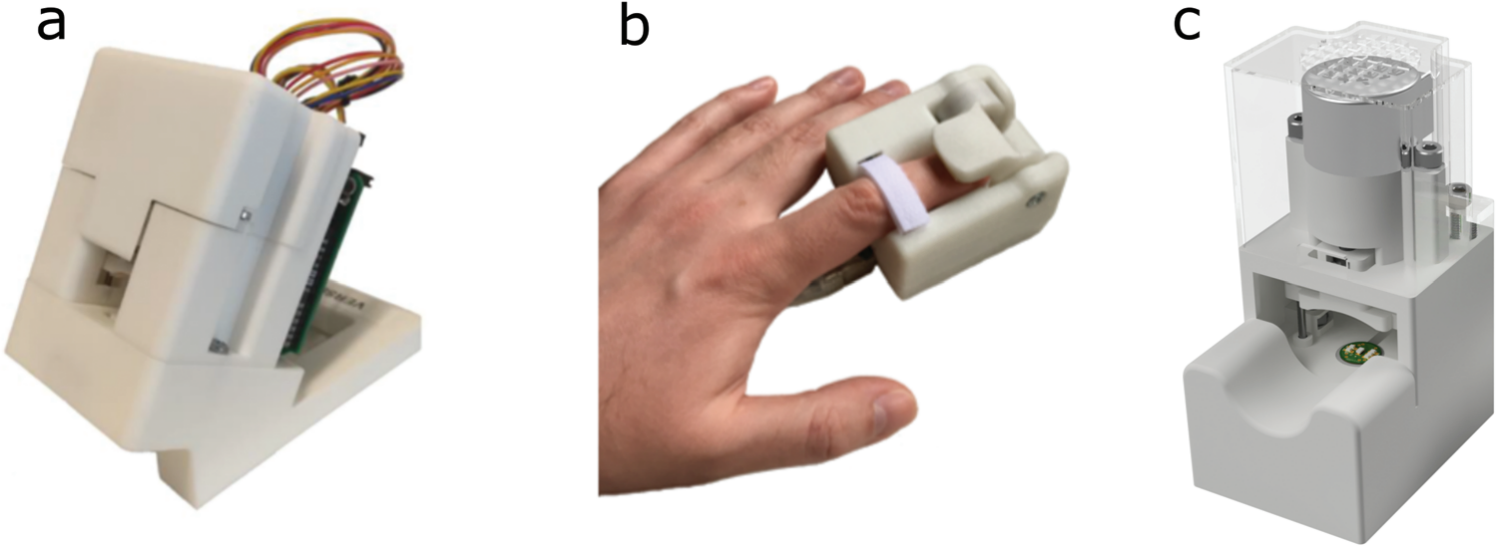
Illustration of used instruments starting from left: a) Instrument I, b) Instrument II, c) Instrument III.

–*Instrument I*: A prototype device had been built for measuring BP and recording arterial pulse waveform. The system consisted of a 3D printed casing that houses the sensor and control electronics. The fingertip was placed on the piston and a force was applied to the opposite side of the finger via a miniature linear motion actuator. The sensor consists of a standard barometric pressure sensor (Bosch Sensortec BMP180), an air cushion, and a cylindrical piston. The protective metal lid of the pressure sensor was pried off to expose the MEMS element. The pressure was read via I^2^C by the MCU (Atmel ATMega328) and sent to a PC via UART. Details are provided in ref. [13], and the photograph of the device is shown in **Figure** **6**a.

*Instrument II*: The second prototype was built using the same sensing module and the sensing principle, but in a miniaturized finger‐worn package. The mechanical construction of the device holds all electrical components required to apply controlled pressure to the fingertip and tonometric pressure sensor module. The device was powered by a USB cable and controlled by a custom software developed in Python. The enclosure was 3D printed using a selective laser sintering (SLS) process. The controlled force directed on top of the finger was achieved using a lever type solution that rotates around a fixed point (instrument I used a linear motion actuator). The end of the lever that makes contact with the finger was made from a flexible 3D printing filament. A DC motor controls the lever actuation. A reduction gear train and a worm gear assembly were also used to further reduce the rotational speed and to change the angle of rotation. Details are provided in ref. [17] and the device photograph is shown in Figure 6b.

*Instrument III*: This version of the instrument extends Instrument I described above, by adding a time‐synced PPG sensor. The PPG sensor consists of a photodiode (Vishay Semiconductors VEMD1060X01) with wide spectral bandwidth (350–1,070 nm, peaking at 820 nm) and five light‐emitting diodes (LEDs) with wavelengths of 465 nm (blue, Kingbright APT1608VBC/D), 515 nm (green, Kingbright APT1608ZGCK), 590 nm (yellow, Kingbright APT1608SYCK/J3‐PRV), 640 nm (red, Kingbright APT1608SECK/J3‐PRV), and 880 nm (infrared, Kingbright APT1608SF4C‐PRV). The custom‐made PPG sensor was mounted on top of the tonometric piston touching the fingertip. The instrument, thus, allows recording simultaneous pressure and multi‐wavelength optical signals. The compression force was created as in Instrument I, except that the pressure sensor was updated to a newer version (Bosch Sensortec BMP280). Additionally, the pressure sensor was not modified by prying the protective metal lid. Instead, the whole sensor PCB was cast in epoxy to level the surrounding area with the top of the metal cover. A small air cushion was sealed around the pressure sensor using a piece of double‐sided tape with strong adhesion creating a closed space. The pressure of this space was then read using the unmodified barometric pressure sensor. More details are provided in ref. [22]. The device is shown in Figure 6c.

All instrument versions share the same fundamental pressure sensing technique. Instrument II was a wearable form factor version of Instrument I and was targeted for continuous long‐term measurements at the bed‐side. The instrument III had a similar table top form factor as Instrument I, but also includes a PPG sensing unit, which the previous versions did not have. The PPG in this study was used to compare the capabilities of these two modalities, but it also allowed direct integration of oxygen saturation measurement into a single device.

### Human Studies

Measurements were carried out according to the Declaration of Helsinki guidelines with the permission of the Ethics Committee of the Hospital District of Southwest Finland and the National Supervisory Authority for Welfare and Health. A written informed consent was provided by each study participant. The trial registration number was NCT04218032 at ClinicalTrials.gov. The studies followed the protocol set by US Association for the Advancement of Medical Instrumentation (AAMI) which was a statement that presents the key aspects of a validation procedure for blood pressure monitors.^[^ ^26^ ^]^ This study did not fully meet all the requirements concerning the number of participants recruited, specified blood pressure range and the used reference device was auscultation in the dataset II and oscillometry in others. The used datasets and instruments are summarized in **Table** **3**.

**Table 3:** Summary of datasets and devices used in this study.

Instrument	Dataset	(n)	Details	Analysis	Refs
I	Dataset I	(33)	Pressure sensor	Oscillometric BP	[13]
II	Dataset II	(43)	Pressure sensor,	Oscillometric BP	[17]
		(SR:43;AF:6)	Miniaturized	Oscillometric AF detection	
III	Dataset IIIa	(10)	Pressure sensor, PPG	Oscillometric BP	[22]
	Dataset IIIb	(SR:14,AF:5)		Similarity analysis	
	Dataset IIIb	(SR:14,AF:5)		Non‐oscillometric AF detection	

*Dataset I*: In the first study, 33 volunteers (weight: µ = 77 kg [range: 48 to 117 kg]; height: µ = 176 cm [range: 161 to 194 cm]; age: µ = 32 [range: 23 to 75 years], 8 women) were measured using the instrument I and a commercially available validated cuff‐based NIBP monitor (Omron Intellisense M6). The subjects had a SBP and DBP ranges of 56 to 84 mmHg and 93 to 146 mmHg, respectively. Four participants were on BP medication. The measurements were taken with the subject in a supine position to ensure that both the finger sensor and the cuff device were at heart level. Subjects were also asked to relax and wait 10 min before the first measurement to ensure that BP was stable. Three measurements were performed on both the reference and the experimental devices from which the average values were calculated.

*Dataset II*: In the second clinical trial, 32 volunteers (age: µ = 47 years [range: 24 to 83 years], 5 women) were recruited for measurements using the instrument II and manual auscultation. One subject was excluded due to severe nail clubbing. Six subjects with persistent AF were excluded from the BP validation dataset (n = 25), but were used to validate the detection of AF. The validation study was carried out at Turku University Hospital. Similarly to the first study, the subjects were placed in a supine position. The finger probe was placed on the index finger and an arm cuff connected to a mercury column sphygmomanometer was placed around the upper arm. Manual auscultation was performed by two trained observers, according to the AAMI standard. After an initial test measurement, three measurements were taken cyclically from each participant, one device after another, both with the finger device and by auscultation.

To broaden the range of BP, nine individuals (age: µ = 48 [range: 25 to 78 years], four women) were recruited to perform a hydrostatic challenge (HC). Four of the subjects were on BP‐lowering medication. BP was measured by using the device and a clinically validated wrist‐worn BP cuff (Omron R2, Japan).^[^ ^27^ ^]^ The wrist cuff used the oscillometric method for the acquisition of BP. Both instruments were worn in the same arm and were held 10 cm below and above heart level, resulting in a total of 18 data points of SBP, MAP, and DBP. This resulted in a change of approximately 15 mmHg between the two levels. Three consecutive measurements were taken for both hydrostatic levels. Three of the subjects measured in this group were also in the auscultated group, but the measurements were taken over six months apart.

The combined data from the clinical studies (dataset I: n = 33, dataset II: n = 25+18) resulted in total of 76 BP pairs (developed device and reference) for validation.

*Dataset III*: This dataset contained oscillometric measurements taken simultaneously with pressure and optical sensors using the instrument III from ten volunteers (mean age 45 ± 19 years, range of [29, 85] years, five women). Reference BP measurements were taken with a cuff‐based NIBP monitor (Omron M3, HEM‐7154‐E). The reference SBP and DBP readings were converted to MAP using the typical formula of: *MAP* = 1/3 × *SBP* + 2/3 × *DBP*.^[^ ^28^ ^]^ Three measurements were taken with both the reference device and the experimental device from each subject in the supine position. Three measurements were omitted due to poor measurement quality, resulting in a total of 27 oscillometric measurements. This dataset is known as IIIa.

Furthermore, arterial waveform signals were recorded with the instrument III from 19 subjects (mean age 58 ± 19 years, range of [29, 84] years), four women) under constant external pressure. Five subjects had persistent AF. One measurement was taken from each subject in a supine position where the external pressure was raised approximately to MAP and held there for about 2 min. This dataset is known as IIIb.

### Data Analysis

Algorithms for BP, heart beat, and AF detection were developed, and a similarity analysis between the pressure and optical sensor recordings were performed. Algorithms were developed in Python and data was analyzed offline post measurement.

*Beat‐to‐beat detection*: Heart beat detection was achieved by finding the foot of each pulse waveform, which was the characteristic point that marks the beginning of a new pulse. Waveform feet were detected from the band‐pass filtered signal (Butterworth filter with cutoff frequencies set at 0.5 and 8 Hz) using the automatic multiscale‐based peak detection (AMPD) algorithm.^[^ ^29^, ^30^ ^]^ The detected feet were visually and independently validated by two annotators.

*Oscillometric BP*: The BP values were extracted from the oscillometric recordings by subtracting the atmospheric pressure from the signal and filtering the signal with a band‐pass filter (1 Hz, 10 Hz). This was followed by a Hilbert transform and polynomial fitting. This was followed by a peak detection by finding local maxima resulting in a bell‐shaped oscillometric waveform envelope (OMWE). The external pressure at which the envelope reaches its maximum was taken as the MAP. The pressure level corresponding to 80% of the maximum oscillations on the lower pressure side of the OMWE was set as DBP.^[^ ^13^ ^]^ SBP was calculated from DBP and MAP using *SBP* = (*MAP* − (1 − *k*) × *DBP*)/*k*. The parameter *k* was computed using two different methods. In the first method (dataset I), *k* was fixed at 0.4 for all subjects. The second approach (dataset II), which was patient‐specific, the arterial stiffness index (ASI) and set *k* = 0.4 + *l* was used, where $l=5({\tilde{x}}_{ASI}-ASI)$. ${\tilde{x}}_{ASI}$ was 0.03 and it is the median of the ASIs calculated in dataset II. Details for computing ASI are given in ref. [17].

The oscillometric BP was estimated from the PPG envelope by finding the external pressure from the pressure signal that corresponds to the MAP of the PPG oscillogram envelope.

*Atrial fibrillation*: The detection capability of AF in continuous pulse waveform measurements (dataset IIIb) was investigated by computing two specific features, pNN50 and area under autocorrelation (AUA).^[^ ^20^ ^]^ The signal was segmented into five‐second epochs and for each epoch, pNN50 and autocorrelation was calculated. These arrays of features were averaged, resulting in one pNN50 value and an ensemble averaged autocorrelation for each recording. The final AUA was calculated from the ensemble average, as in ref. [20].

Envelope error for detecting AF during oscillometric measurements (dataset II) was computed for all subjects. The mean error from oscillometric waveform envelope in mmHg was computed by subtracting the individual pulse amplitudes from the envelope and averaging over the whole measurement. In AF, the pulse pressure varies randomly, resulting in deformation of the oscillometric envelope. Single pulses could be higher or lower than the OMWE, increasing the mean error.

*Correlation analysis*: Correlation analysis was carried out in two ways for the dataset III; i) corresponding waveforms, one waveform per cardiac cycle, were detected in each sensor channel (red, infrared, and pressure) in dataset IIIb. Then, for each channel pair combination(red & infrared, red & pressure, and infrared & pressure) an average Pearson correlation coefficient was calculated. This correlation was computed for each channel pair, by computing a correlation coefficient for each matching waveform in the channels resulting in a array of correlation coefficients that were subsequently averaged. This was repeated for each measurement. ii) The similarity of oscillogram envelopes was evaluated in a similar way for dataset IIIa. Oscillogram envelopes, one for each recorded channel (red, infrared, and pressure) per measurement were extracted as previously described. Then, for each channel combination pair, a Pearson correlation coefficient was computed for each measurement.

### Statistical Analysis

The recorded signals were pre‐processed using filters detailed in Section Data Analysis. The sample size for each analysis is detailed in Section Human Studies. Blood pressure accuracy was presented through correlation plot with correlation coefficient (r), Bland‐Altman plot with mean difference and ± 1.96 interval and summary results were presented in a table with (mean ± SD) over the cohorts. The box plot lines and whiskers for the features in atrial fibrillation analysis present the median and full range of data. No outliers were excluded in any of the analyses. Data analysis was performed using Python 3.

## Conflict of Interest

The authors declare no conflict of interest.

## Supporting information

Supporting Information

## Data Availability

The data that support the findings of this study are available on request from the corresponding author. The data are not publicly available due to privacy or ethical restrictions.
