# Supplementary material for: Hemodynamic Bedside Monitoring Instrument with Pressure and Optical Sensors: Validation and Modality Comparison
Source: Adv Sci (Weinh). 2024 Apr 22;11(24):2307718. doi: 10.1002/advs.202307718 (PMC11200005; doi:10.1002/advs.202307718)
Supplement: Supplementary file 1 — Supporting Information [file ADVS-11-2307718-s001.pdf]

ADVANCED SCIENCE
Open Access

Supporting Information

for *Adv. Sci.*, DOI 10.1002/advs.202307718

Hemodynamic Bedside Monitoring Instrument with Pressure and Optical Sensors: Validation and Modality Comparison

Matti Kaisti, Tuukka Panula, Jukka-Pekka Sirkiä, Mikko Pänkäälä, Tero Koivisto, Teemu Niiranen and Ilkka Kantola*

WILEY-VCH

Supporting Information: Hemodynamic Bedside Monitoring Instrument with Pressure and Optical Sensors: Validation and Modality Comparison

Pressure-volume model

A Python implementation of the model is available at https://github.com/mkaist/oscillometry-model.

SI table 1. Oscillometric pressure volume model equations

**Parameters**		
t	time	
r	rate of cuff deflation	
P_0	inflation pressure at beginning	
V_0	baseline cuff volume	
$V_0 - \Delta V_a$	describes cuff volume modulation caused by arterial pulsation	
V_{a0}	arterial volume at zero pressure	
a, b	compliance constants	
PP	pulse pressure	
DBP	diastolic blood pressure	
**Equations**		
$P_t = P_a - P_{ext},\ \text{where } P_{ext} = P_0 - rt$	transmural pressure	(1)
$P_a = DBP + 0.5PP + 0.36PP \times [sin(\omega t) + 0.5sin(2\omega t) + 0.25sin(3\omega t)]$	arterial pressure waveform	(2)
$\frac{dP_a}{dt} = 0.36PP\omega \times [cos(\omega t) + cos(2\omega t) + 0.75cos(3\omega t)]$	time derivative of arterial pressure waveform	(3)
$\frac{dV_a}{dt} = aV_{a0}\exp(aP_t) \times \left(\frac{dP_a}{dt} + r\right)$	time derivative of arterial volume for$P_t < 0$	(4a)
$\frac{dV_a}{dt} = aV_{a0}\exp(-bP_t) \times \left(\frac{dP_a}{dt} + r\right)$	time derivative of arterial volume for$P_t \geq 0$	(4b)
$\frac{dP}{dt} = -r + \left(\frac{P_{ext} + 760)}{V_0}\right)\frac{dV_a(t)}{dt} + P_0$	time derivative of cuff pressure	(5)
$P(t) = \int_0^t \frac{dP}{dt}\, dt$	cuff pressure	(6)

Pressure coupling of the barometric sensor

The force exerted on top of the finger propagates through the piston to the air cushion. The force is equal in each boundary of this stacked structure. The relation between pressure (P) and force (F) is $P = F/A$ and thus the pressure at the boundaries is dictated by their respective surface areas (A).
As the area on top of the piston and the air cushion are approximately equal, the pressures on both sides of the cushion wall are also equal. The fixed walls and floor of the cylinder prevent the cushion from expanding under external pressure. Since pressure applied to a fluid is evenly exerted throughout the fluid, the pressure 'seen' by the barometric sensor die by the cushion is also equal to the pressure applied to the finger (P_{piston}). The relationship can be expressed as

$$P_{piston} = P_{cushion} \iff \frac{F_{piston}}{A_{piston}} = \frac{F_{cushion}}{A_{cushion}}, \quad (1)$$

where $P_{cushion}$ is the pressure inside the air cushion, F_{piston} and $F_{cushion}$ are the forces exerted to the finger and the cushion respectively and A_{piston} and $A_{cushion}$ are the corresponding areas.
The validation of the applied pressure coupling to the sensing element is confirmed by placing known weights on top of the sensor and comparing measured pressure values to those obtained by the equation

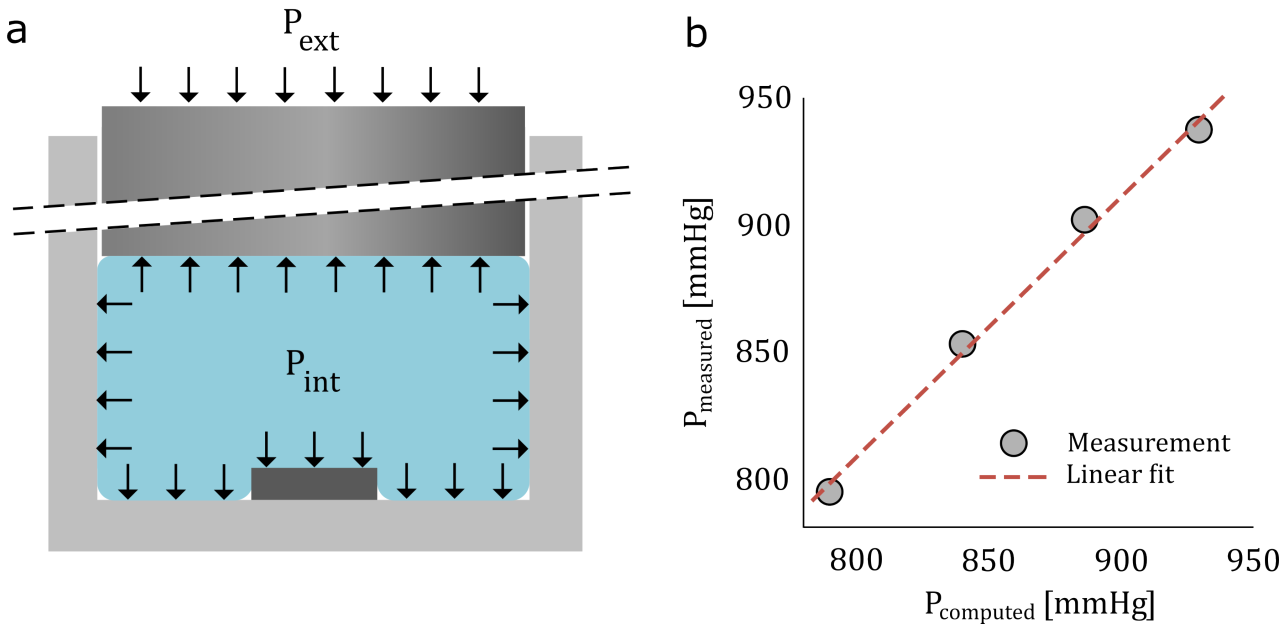

SI Fig. 1. a) Coupling of pressure from piston surface to barometric sensor. b) Measured pressure response compared against computed pressure at a range approx. 30 to 180 mmHg with the measured atmospheric pressure subtracted).

$P_{cushion} = F_{piston}/A_{piston}$. The sensor's measured response aligns well with the predicted values, exhibiting a linear response as shown in Figure 1.

At the presence of arterial pulse the P_{piston} is a sum of two pressures, P_{aeff} and P_{ext} where P_{aeff} is effective pressure oscillation caused by the pulsating artery pressure (P_a) and P_{ext} is the external pressure applied to the finger. The effect of pulsating artery when extracting the external pressure during oscillometric measurement is averaged out. The force (F_a) generated by the pulsating applanated artery is relatively small due to the large contact area with the piston surface (A_{piston}) compared to the small artery area (A_a). The system is also insensitive to the P_{aeff}, which can vary due device and artery geometry, since the oscillometric measurement only measures the value of P_{ext} when P_{aeff} is largest.
